# Parenting Styles and Suicidal Behaviors among College Students: Examining the Mediating Roles of Coping, Self-Esteem, and Depression

**DOI:** 10.3390/bs14080666

**Published:** 2024-08-01

**Authors:** Ruishen Liu, Qun Zhao, Shengchang Li, Hongyu Gui, Tianyu Zhang, Jie Wang, Jing Sui

**Affiliations:** 1 Research Institute for Environment and Health, Nanjing University of Information Science and Technology, Nanjing 210044, China; 202113110040@nuist.edu.cn (R.L.); 20201124005@nuist.edu.cn (H.G.); suijing@nuist.edu.cn (J.S.); 2 Institute for Artificial Intelligence in Medicine, Nanjing University of Information Science and Technology, Nanjing 210044, China; 202083460065@nuist.edu.cn; 3 School of Management Science and Engineering, Nanjing University of Information Science and Technology, Nanjing 210044, China; 202013630102@nuist.edu.cn; 4 School of Mathematics and Statistics, Nanjing University of Information Science and Technology, Nanjing 210044, China; 202213870060@nuist.edu.cn; 5 Key Laboratory of Environmental Medicine Engineering, Ministry of Education, School of Public Health, Southeast University, Nanjing 210009, China

**Keywords:** parenting styles, coping, self-esteem, depression, suicidal behaviors

## Abstract

Background: Suicide is influenced by multiple factors. However, the mechanisms through which these factors influence suicide remain understudied. This study aims to examine the relationship between parenting styles (warmth, control, indulgence, humiliation, and neglect), coping, self-esteem, depression, and suicidality (suicidal ideation and suicide attempts) among college students. Methods: Cross-sectional data were collected from 2369 undergraduates (mean age = 20.10 years) including 1201 women (50.7%) at four Chinese colleges. Results: Students reported high rates of suicidal behaviors (12.7% suicidal ideation, 6.4% suicidal attempts) and depression (37%). Structural equation modeling indicated that warmth (+) had associations with coping. Coping was linked to self-esteem and depression. Depression (+), self-esteem (−), warmth (−), and neglect (+) had direct correlations with suicidality. Self-esteem mediated the relationships between warmth and depression. Conclusions: Future prevention intervention efforts aimed at reducing depression and suicidal behaviors should prioritize the promotion of positive parenting styles and the avoidance of negative ones. College mental health services should emphasize positive and optimistic coping strategies to enhance students’ self-esteem.

## 1. Introduction

The World Health Organization estimated in 2021 that over 700,000 people died by suicide each year globally, with the suicide rate reaching over 1‱ among people aged 15–29 years. Furthermore, suicide is the fourth leading cause of death in the adolescent population [1]. Severe depression and anxiety have been found to be significant contributors to suicidal behavior [2].

Research has indicated that many individuals who contemplate suicide due to depression often experienced a lack of parental care and attention during their upbringing. They are at increased risk of mental health challenges and adverse outcomes such as poor academic performance, employment-related stress, and societal stigma [3]. Becker et al. [4] reported that these factors can precipitate a social psychological crisis without effective interventions, potentially leading to suicidal ideation and attempts. Previous studies have determined that variations in parenting style attributes, such as parental attitudes, control, rejection, absence of democracy, and overprotection in parent–child relationships, significantly affect children’s personality traits and their overall mental health status [5,6,7]. McCoby et al. [8] introduced a four-dimensional model in 1983, encompassing authoritative, authoritarian, permissive, and neglectful parenting. This model offers a deeper understanding of parenting styles and their intrinsic factors. In addition, Skinner et al. [9] added to the parenting style model, (e.g., warmth, rejection, structure, chaos, autonomy support, and coercion). A warmth parenting approach not only fosters children’s mental health but also has a beneficial effect on their physical health [10]. Wu et al. [11] discovered that a warmth parenting style positively correlates with children’s perceived life satisfaction but is inversely related to mental disorders. Thus, democratic, warm, and responsive parenting methods play a pivotal role in enhancing children’s cognitive flexibility and subjective well-being. Additionally, Lepe et al. [12] noted that in regions with underdeveloped social education and support, a warmth parenting style could notably help to reduce negative mental health indicators in children (e.g., depression, anxiety). Conversely, neglectful parenting not only impedes the development of children’s accurate cognition but also hinders their social adaptability.

Coping is a crucial psychological factor influencing suicide. According to Eisenberg et al. [13], coping pertains to an individual’s self-regulation when confronted with stress, embodying the process of managing emotions and behaviors. Broadly speaking, coping is categorized into positive and negative types, according on outcomes from academic discussions. Positive coping is further divided into positive cognition and positive behavior, whereas negative coping is characterized by thoughts and behaviors that lean toward avoidance when dealing with challenges [14]. Research on coping has a longstanding history. Finstad et al. [15] determined that negative coping styles have a strong association with stressful occurrences, stressors, and traumatic episodes. Thus, adopting positive and effective coping methods is pivotal for individuals to cope with depression and deter high-risk behaviors. Furthermore, earlier research indicated that without adequate coping mechanisms, these mental challenges can usher in psychosocial issues, potentially culminating in suicidal thoughts and actions [16,17].

Self-esteem profoundly affects suicidal tendencies. As defined by [18], the executive director of the National Association for Self-Esteem, self-esteem embodies an individual’s life-affirming sentiment and alignment with life’s demands, coupled with their confidence in overcoming diverse life challenges and their entitlement to a brighter future. A plethora of research underscores the significant influence of individual self-awareness factors (like self-reflection, self-assessment, and self-expectation), as well as self-esteem and self-confidence, on the extent of a person’s depression. Davidson [19] posited that self-esteem acts as a barometer for gauging individual depression levels, suggesting that a person with diminished self-assurance is more prone to profound depression. Additionally, an empirical analysis by Sowislo et al. [20] demonstrated that the level of self-esteem is a more accurate predictor of a person’s depressive symptoms than the stability of their self-esteem.

Young adults, especially college students, encounter numerous challenges, including forming and maintaining new relationships, managing escalating academic pressures, and navigating perceived limited job opportunities [21]. These challenges can heighten the risk of depression in this population, with extreme cases progressing to severe depression, self-harm, or suicide. In a survey, Gao et al. [22] reported that over half the college students at Peking University exhibited signs of depression, with more than a third being mildly depressed and nearly a fifth being moderately or severely depressed. Li et al. [23] determined that cumulative stress events, depression, sleep issues, social challenges, and pervasive feelings of despair are strongly linked to suicidal behavior. These tendencies, however, can be considerably mitigated with support from family and friends and by fostering feelings of hope and attention from others. To gain a deeper understanding of the factors influencing suicidal behavior in young populations, especially college students, this research focused on college students to identify the primary determinants of their suicidal behaviors.

Previous studies have frequently utilized analytical methods such as correlation and regression. For instance, Chen et al. [24] applied correlation and logistic regression analyses to assess the effects of individual, familial, and social factors on suicidal ideation (SI), suicide attempts, (SA), and attempted suicide (AS) among adolescents. Their findings indicated that familial and social factors are pivotal in preventing and intervening in adolescent suicide. Tang et al. [25] used logistic regression to estimate the impacts of stressful life events and coping skills on the risk of suicidal behaviors among Chinese college students. They concluded that stressful life events and inadequate coping skills are significant risk factors leading to suicidal behaviors in adolescent students. Additionally, some researchers have investigated college students’ suicidal or depressive tendencies using mediator models and regulation models. Yet, the selection of mediator variables has typically focused on the mediating or regulating effect of a singular variable. In a study examining the relationship between bullying and medication use among 419 college drinkers, Luk et al. [26] employed a mediation model within a structural equation modeling framework. Their research discovered that the authority of both mothers and fathers can deter bullying and depression by enhancing self-esteem. In another study probing the influence of self-esteem on mindfulness, anxiety, and depression among 417 undergraduate students, Bajaj et al. [27] designed a moderation model with self-esteem serving as the moderating variable. They established that self-esteem can help to reduce anxiety and depression in the context of mindfulness.

In summary, the majority of past research did not incorporate multiple variables as mediators or moderators when investigating the effects of suicide among college students. Consequently, this study aimed to employ the structural equation modeling technique to scrutinize the mediating impacts of three variables, self-esteem, coping, and depression, on the relationship between college students’ suicidal behaviors and parenting styles, as well as the direct influences of parenting styles.

### Research Hypotheses and Questions

Firstly, previous studies have shown that parenting styles could be directly correlated with suicidal behaviors [12,24]. Therefore, we posited that parenting styles predict suicidal behaviors. Secondly, as evidenced by Colmenero et al. [28] and Olatunji and Idemudia [29], parenting styles can be indirectly related to suicidal behaviors through self-esteem and depression. Hence, we hypothesized that self-esteem and depression predict suicidal behavior as mediators, but we did not specify a positive or negative direction. Built on the two hypotheses, we tested whether coping plays a mediating role between parenting styles, self-esteem, and depression.

## 2. Materials and Methods

### 2.1. Participants and Procedure

Data for this study were sourced from college students in China from 2017 to 2018. Two universities and two vocational–technical colleges in a large, economically developed city in eastern China were randomly chosen for participation to ensure the data’s scientific rigor and comprehensive nature. Informed consent was secured from participants, parents, and schools for all procedures. To garner more substantial support from schools, we promised individual psychological feedback to each class as a whole and to the students before the questionnaire distribution, along with potential future collaborations on interventions. Our study sample was chosen through stratified whole-group sampling based on school grade. From the four grades (i.e., freshman, sophomore, junior, and senior), 4–10 classes were randomly picked, and all students in these classes completed the questionnaires. In total, we collected data from 2419 students. After discarding invalid questionnaires, we had 2369 valid ones, representing a 98% valid response rate. Of these, 1168 (49.3%) were from male participants, and 1201 (50.7%) were from female participants. The mean age was 20.10 years (SD = 1.24). Among the sample, freshmen accounted for the largest proportion, with male students outnumbering women in this subgroup (57.8% vs. 48.3%; *p* < 0.001). In contrast, there was only a small proportion of students in their junior year and above. Approximately 13.6% of college students had been involved in suicidality, with similar percentages for men and women. Table 1 provides more detailed information. The questionnaire took about 20–30 min to complete. Participants filled out the questionnaire independently, and, as a token of appreciation for their involvement, each was given either a notebook or a pen after completing the survey. The study protocol and consent procedures received approval from the Institutional Review Board (IRB) of Zhongda Hospital Southeast University (2021ZDKYSB211), Nanjing, China.

### 2.2. Measures

#### 2.2.1. Sociodemographic Characteristics

The sociodemographic variables included in our analyses were age (in years), sex (male or female), and grade (freshman, sophomore, or junior and above).

#### 2.2.2. Suicidality

Participants were asked to answer two questions: (1) “Did you seriously think about committing suicide during the last year?” (suicidal ideation), and (2) “Did you ever try or attempt to kill yourself during the past year?” (suicide attempts). These two items were rated on a 5-point scale (1 = never; 2 = seldom; 3 = occasionally; 4 = sometimes; and 5 = very often). For the purpose of analysis, suicidality (ideation or attempts) was dichotomized into “no” (response of “never” to both questions) and “yes” (response of any other option to either question). The internal consistency for suicidality was 0.82 for the current sample.

#### 2.2.3. Depression

Depression was measured using the Center for Epidemiological Studies Depression Scale [30]. It demonstrated high internal consistency of 0.87 and satisfactory test–retest repeatability. The total CES-D score ranges from 0 to 60, with higher scores indicating more frequent depressive symptoms, which correspond to a more severe depressive condition. A score below 16 suggests no depression, a score of 16–21 signifies mild depression, and a score above 21 denotes major depression.

#### 2.2.4. Self-Esteem

Self-esteem was assessed using the Rosenberg Self-Esteem Scale [31]. This scale consists of 10 items, aiming to evaluate adolescents’ overall feelings about self-worth and self-acceptance. Each item was scored on a five-point Likert scale. Higher scores indicate a more robust sense of personal self-esteem: scores below 15 suggest a weaker sense of self-esteem, scores from 15 to 25 reflect an average level of self-esteem, and scores above 25 denote a stronger sense of self-esteem. The internal consistency for self-esteem was 0.67.

#### 2.2.5. Coping

Coping style was gauged using the Simplified Coping Style Questionnaire (SCSQ) [32]. This scale comprises 20 items. Each item was ranked from 0 (never) to 4 (always), indicating the significance of different coping styles. A higher composite score represents a more effective coping style. The internal consistency for coping was 0.84.

#### 2.2.6. Parenting Styles

The 13-item Chinese version of the Egna Minnen av Barndoms Uppfostran (EMBU-CV) was employed to measure parenting styles in this study [33,34]. Items were split into five subscales: “warmth” (e.g., “I felt loved by caregivers”), “control” (e.g., “caregivers interfered with everything I did when I was a child”), “indulgence” (e.g., “caregivers played favorites with me when I caused trouble”), “humiliation” (e.g., “caregivers often beat up/rebuked/embarrassed me”), and “neglect” (e.g., “caregivers had no time to take care of me or give me company”). Each item was scored on a 4-point scale: 1 = never, 2 = occasionally, 3 = sometimes, and 4 = often. The internal consistencies of the EMBU-CV ranged from 0.60 to 0.73.

### 2.3. Data Analysis

This study employed structural equation modeling (SEM) as the primary data analysis approach to explore the intricate relationships among parenting styles, self-esteem, depression, coping strategies, and suicidality. The constructed equation model was examined using AMOS 21.0 software, which performed parameter estimation of the initial structural model, model fitting, model evaluation, and model correction. The structural equation model has numerous statistical indicators. In order to check the overall model fit, CMIN/DF, GFI, CFI, NFI, IFI, TLI, and RMSEA were selected. By convention, CMIN/DF should be at least less than 5 and preferably less than 3 or 2; GFI, NFI, IFI, TLI, and CFI should be greater than 0.90, where the closer to 1, the better; RMSEA should be less than 0.05, where the closer to 0, the better. When evaluating the fit of the model to the data, all indicators should be considered. Only when most of the indicators meet the conventional requirements is the model considered to be a good fit to the data, and the model is accepted.

## 3. Results

### Group Differences in Demographic Characteristics

Table 1 delves deeper into the nuances of sex differences among our college student participants. By measuring the scores of depression (using CES-D), self-esteem, and coping scales among college students, it could be concluded that depression was more severe among men (19.83 vs. 17.58; *p* < 0.001). However, there were no statistically significant differences in the strength of self-esteem between men and women, but women were more capable of coping (51.11 vs. 52.96; *p* < 0.001). Parenting styles were classified into five categories: warmth, control, indulgence, humiliation, and neglect. College students identified differently with different parenting styles. The scores for control (2.58 vs. 2.34; *p* < 0.001), indulgence (1.93 vs. 1.66; *p* < 0.001), humiliation (1.81 vs. 1.57; *p* < 0.001), and neglect (2.02 vs. 1.78; *p* < 0.001) were all higher for men than for women, while the scores for warmth (3.25 vs. 3.38; *p* < 0.001) were higher for women than for men.

The bivariate correlations of the indicator variables are presented in Table 2. Most of the correlation coefficients were statistically significant at the *p* < 0.05 level. There was a significant positive correlation between the indicator variables of the same underlying construct (*p* < 0.001).

**Figure 1:**
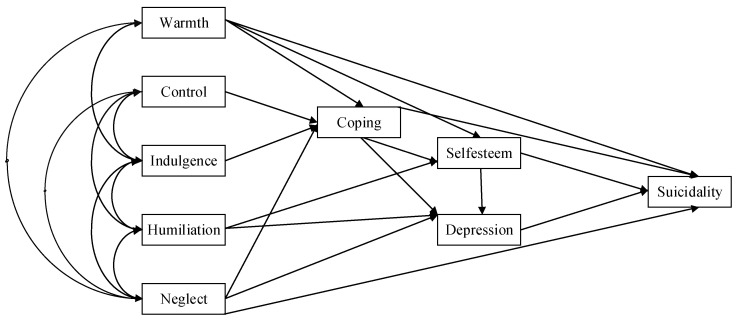
Hypothesized model of suicidality, depression, self-esteem, coping, and parenting styles.

The initial model assumptions are depicted in Figure 1. The corresponding indicator variables and latent variables are also included in Figure 1. Thus, in total, three potential variables and six indicator variables are presented in Figure 1.

**Figure 2:**
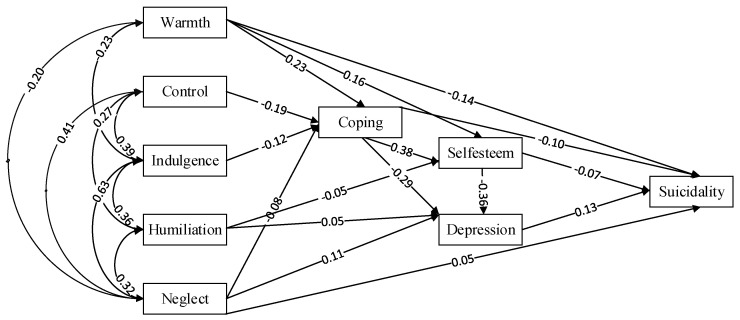
Measurement and structural model for parenting styles and suicidality. Standardized factor loadings appear on the single-headed arrows connecting the latent and observed variables.

The modified measurement model had a good fit, with CMIN = 19.595, DF = 12, CMIN/DF = 1.633, GFI = 0.998, NFI = 0.996, CFI = 0.998, and RMSEA = 0.016. The final structural equation model fitted the data well (Figure 2).

## 4. Discussion

This study aimed to examine the roles of five parenting styles: warmth, control, indulgence, humiliation, and neglect, in the formation of suicidality in adolescents and to analyze the mediating roles of coping, self-esteem, and depression in the relationship between parenting styles and suicidality. In examining the relationship between parenting styles and suicidality, we found that warmth parenting demonstrated a significant protective effect and was associated with a lower risk of suicidality. In contrast, the other four parenting styles were associated with the occurrence of suicidality to varying degrees, with neglectful parenting in particular having the most significant positive effect on suicidality. The three factors of coping, self-esteem, and depression were found to significantly mediate the relationship between parenting styles and suicidality, with coping and self-esteem having a negative effect on suicidality, and depression having a positive effect on suicidality.

Similar to previous studies, this study found a significant correlation between parenting styles and suicidality in adolescents [35,36]. In the current study, parenting styles were divided into five categories, namely warmth, control, indulgence, humiliation, and neglect, which was different from the division according to the four-quadrant model in previous studies, which explored neglectful/uninvolved parenting, authoritarian parenting, permissive parenting, and authoritative parenting [37,38,39]. The current study more accurately measured the versatility and complexity of modern parenting styles. García and Gracia [40] pointed out that the indulgence parenting style was a distinguishing feature in the assessment of parenting styles, suggesting that our measure makes up for the limitations of previous categorizations.

We found that all the parenting styles that had a positive effect on suicidality were directly or indirectly associated with the development of depressive symptoms. Among the three mediating factors, depression was also the one that had a relatively significant effect on suicidality. We also found significant associations between parenting styles and depression, with different types of parenting styles affecting depression to varying degrees, which is consistent with the findings of Ebrahimi et al. and Milevsky et al. [41,42]. Of the five types of parenting styles, the humiliation and neglect styles had the most significant association with depression, and they influenced suicidality primarily through depression as a mediator. Although much of the literature, such as Handley et al. [43], suggests that depression is a significant contributor to suicidal ideation, Levy and Deykin [43] provided a different idea that almost half of the students who admitted to having made a suicide attempt did not meet the diagnostic criteria for major depression at any time in their lives. This strong correlation can be explained in part by Berkson’s bias [44]; that is, people who suffer from two or more diseases are more likely to be hospitalized than those who suffer from only one disease. Therefore, in a clinical setting, people who are suicidal but not depressed are not as likely to be detected as people who have both suicidal behaviors and depression. It also follows that suicide may manifest itself in symptoms other than depression, such as the low self-esteem and inadequate coping skills in this study.

Aremu et al. [45] identified a significant correlation between parenting styles and adolescent self-esteem. Our research indicated that self-esteem, acting as a mediator, was directly linked to the warmth parenting style and indirectly to other parenting styles, primarily through coping mechanisms. Furthermore, self-esteem had both a direct and indirect influence on suicidal behaviors, the latter being mediated by depression. Orth and Robins [46] established a notable connection between self-esteem and depression, suggesting that lower self-esteem levels, rather than instability or over-reliance on self-esteem, played a pivotal role in exacerbating depression. This susceptibility to depression is predominantly influenced by general self-esteem. Such an effect can manifest in two ways: global self-esteem, where individuals with diminished self-worth resist positive influences and perceive undesirability in positive outcomes [47], and domain-specific self-esteem, where individuals harbor negative perceptions of themselves in specific areas, including academic performance, social competence, and physical appeal. Both perspectives can potentially foster depression. In a recent study by Klonoff-Cohen [48], approximately one-third of college students advocated for bolstering self-esteem as the paramount approach to curb self-harm and suicidal tendencies. Notably, amid the COVID-19 pandemic, the emergent social isolation among college students led to a decline in self-esteem, underscoring the imperative of nurturing positive self-worth to safeguard mental health and diminish self-harm and suicide risks [49].

The effect of coping as a mediating factor appears more intricate than that of other factors. Four parenting styles (with the exception of humiliation) significantly influenced coping. However, only the warmth parenting style had a positive effect on coping. Given the current Chinese social context, parents might lean toward the indulgence parenting style, especially when raising an only child, leading to overprotectiveness. This could render these children ill-equipped with the necessary coping mechanisms to navigate the pressures and challenges of college upon admission [50]. Our research further highlighted a significant link between coping and suicidality, corroborating earlier studies in both Western and Chinese adolescents which suggested that positive coping strategies are inversely related to suicidality [51,52]. Zhang et al. [53] indicated that college students, much like children and early-to-middle adolescents, exhibited unstable coping abilities because these skills were still maturing. Thus, fostering coping skills during college is paramount. Additionally, the salience of coping is manifested in its significant interplay with other mediators, directly influencing self-esteem and depression. Our findings reveal that effective coping mechanisms not only bolster self-esteem in young individuals but also diminish the likelihood of depression. This aligns with Goodwill [54], who noted that inadequate coping tactics, such as “behavioral disengagement” and “self-blame,” correlate with diminished self-esteem levels.

Based on McLoughlin et al.’s study [55], the escalating suicide rates among adolescents globally highlights the urgent need for implementing age-specific measures to curb these behaviors. Therefore, another objective of our research was to devise more targeted interventions to mitigate suicidal behaviors by delineating the mechanisms through which various parenting styles impact children’s propensity for these behaviors. Warmth parenting predominantly embodies the extent to which parents acknowledge and respond to their children’s actions [56]. Research by Hibbard and Walton [57] suggests that elevated parental warmth fostered a nurturing household atmosphere, empowering individuals to undertake challenging endeavors without the looming dread of failure and to establish realistic personal benchmarks. Such an environment significantly augments a child’s resilience and self-worth. Consequently, it is imperative for communities and educational institutions to advocate for the adoption of the warmth parenting approach to stave off suicidal tendencies among the youth. Of all the potentially negative parenting styles, neglect parenting has the most pronounced impact on suicidality. Our study also identified similarities in the effects of control and indulgence parenting styles on suicidality. Both styles influence suicidal behaviors indirectly through the mediating factor of coping, rather than directly. According to Gorostiaga et al. [58], parental oversight, when expressed as harsh control—specifically physical and verbal punishment—may undermine a child’s adaptability. This adaptability is crucial for a child’s ability to cope upon entering college. A previous study [59] found that the indulgence parenting style might render children excessively reliant on parental guidance. This implies that college mental health services should emphasize positive and optimistic coping strategies to enhance students’ self-esteem.

Our study had several limitations. First, several studies have suggested that variations in parenting styles can elevate suicide risks [59,60]. This indicates that we might need to assess the impact of each parenting style on mental health individually. Second, our research is grounded in self-reported data, which carries potential pitfalls like recall bias and social desirability bias. For example, the warmth parenting style originated from respondents recalling memories. In addition, participants might either understate or overstate their suicidal thoughts and actions. They might also portray what they deem to be more “acceptable” parenting, influenced by societal norms. Thirdly, the CES-D is used to measure the frequency of depressive symptoms rather than actual severity of depression, which is still arguable. Moreover, parenting style is a dynamic process, whereas questionnaire measures can only obtain cross-sectional data. Last but not least, it is crucial to note that, while influential, parenting styles are not the sole determinant of adolescent suicide. Complex interactions with substance abuse, life expectancy, and genetic factors must also be considered [55].

## 5. Conclusions

Future prevention intervention efforts aimed at reducing depression and suicidal behaviors should prioritize the promotion of positive parenting styles and the avoidance of negative ones. College mental health services should emphasize positive and optimistic coping strategies to enhance students’ self-esteem.

## Figures and Tables

**Table 1:** Individual characteristics of sample.

N (%)	Total	Male	Female	F/t-Value	*p*-Value
Mean (SD)					
Sex	2369 (100%)	1168 (49.3%)	1201 (50.7%)		
Age (years)	20.10 (1.24)	20.16 (1.27)	20.04 (1.20)	5.36	0.02
Grade					
Freshman	1255 (53%)	675 (57.8%)	580 (48.3%)	28.91	<0.001
Sophomore	761 (32.1%)	358 (30.7%)	403 (33.6%)		
Junior and above	353 (14.9%)	135 (11.6%)	218 (18.2%)		
Suicidality					
Never	2048 (86.4%)	1001 (85.7%)	1047 (87.2%)	3.6	0.46
Seldom	213 (9.0%)	105 (9.0%)	108 (9.0%)		
Occasionally	52 (2.2%)	29 (2.5%)	23 (1.9%)		
Sometimes	26 (1.1%)	14 (1.2%)	12 (1.0%)		
Often	30 (1.3%)	19 (1.6%)	11 (0.9%)		
Depression (CES-D)	18.69 (9.27)	19.83 (9.33)	17.58 (9.07)	35.43	<0.001
No (<16)	997 (42.1%)	435 (37.3%)	562 (46.8%)	35.89	<0.001
Mild (16–21)	487 (20.6%)	227 (19.5%)	260 (21.6%)		
Overt (>21)	884 (37.3%)	505 (43.3%)	379 (31.6%)		
Self-esteem	18.07 (3.93)	18.02 (4.11)	18.12 (3.75)	0.35	0.55
<15 Low	337 (14.2%)	175 (15.0%)	162 (13.5%)	6.91	0.03
15–25 Normal	1911 (80.7%)	921 (78.9%)	990 (82.4%)		
>25 High	121 (5.1%)	72 (3.0%)	49 (4.1%)		
Coping	52.05 (7.86)	51.11 (8.39)	52.96 (7.21)	32.92	<0.001
Parenting styles					
Warmth	3.32 (0.67)	3.25 (0.70)	3.38 (0.63)	21.41	<0.001
Control	2.46 (0.73)	2.58 (0.72)	2.34 (0.72)	63.14	<0.001
Indulgence	1.79 (0.77)	1.93 (0.81)	1.66 (0.70)	71.23	<0.001
Humiliation	1.69 (0.74)	1.81 (0.78)	1.57 (0.67)	65.05	<0.001
Neglect	1.90 (0.70)	2.02 (0.72)	1.78 (0.66)	74.7	<0.001

**Table 2:** Correlation coefficients of parenting styles and suicidal behaviors.

Variable	1	2	3	4	5	6	7	8	9
1. Suicidality	-								
2. Depression	0.26 ^c^	-							
3. Self-esteem	−0.22 ^c^	−0.51 ^c^	-						
4. Coping	0.25	0.48 ^c^	−0.43	-					
Parenting styles									
5. Warmth	−0.22 ^c^	−021 ^c^	0.26 ^c^	−0.27 ^c^	-				
6. Control	0.01	0.14 ^c^	−0.08 ^c^	0.12	0.05 ^a^	-			
7. Indulgence	0.08 ^c^	0.17 ^c^	−0.09 ^c^	0.27 ^c^	0.02	0.27 ^c^	-		
8. Humiliation	0.14 ^c^	0.24 ^c^	−0.16 ^c^	0.30 ^c^	−0.22 ^c^	0.36 ^c^	0.39 ^c^	-	
9. Neglect	0.15 ^c^	0.26 ^c^	−0.17 ^c^	0.28	−0.19 ^c^	0.31 ^c^	0.41 ^c^	0.63 ^c^	-

^a^ *p* < 0.05; ^c^ *p* < 0.001.

## Data Availability

The data that support the findings of this study are available from the corresponding author upon reasonable request.
